# Assessment of the MARTINI 3 Performance for Short Peptide Self-Assembly

**DOI:** 10.1021/acs.jctc.3c01015

**Published:** 2023-12-19

**Authors:** Ivan R. Sasselli, Ivan Coluzza

**Affiliations:** † Centro de Física de Materiales (CFM), CSIC-UPV/EHU, Paseo Manuel de Lardizabal 5, 20018 San Sebastián, Spain; ‡ Center for Cooperative Research in Biomaterials (CIC biomaGUNE), Basque Research and Technology Alliance (BRTA), Paseo de Miramón 182, 20014 Donostia-San Sebastián, Spain; § Ikerbasque, Basque Foundation for Science, Plaza de Euskadi 5, 48009 Bilbao, Spain; ∥ BCMaterials, Basque Center for Materials, Applications and Nanostructures, UPV/EHU Science Park, 48940 Leioa, Spain

## Abstract

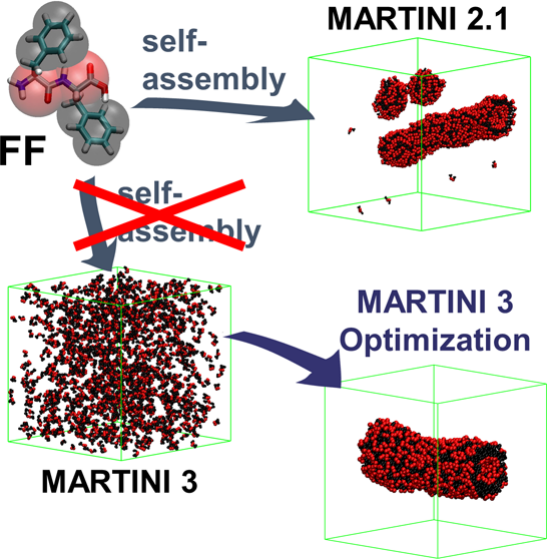

The coarse-grained MARTINI force field, initially developed for membranes, has proven to be an exceptional tool for investigating supramolecular peptide assemblies. Over the years, the force field underwent refinements to enhance accuracy, enabling, for example, the reproduction of protein–ligand interactions and constant pH behavior. However, these protein-focused improvements seem to have compromised its ability to model short peptide self-assembly. In this study, we assess the performance of MARTINI 3 in reproducing peptide self-assembly using the well-established diphenylalanine (FF) as our test case. Unlike its success in version 2.1, FF does not even exhibit aggregation in version 3. By systematically exploring parameters for the aromatic side chains and charged backbone beads, we established a parameter set that effectively reproduces tube formation. Remarkably, these parameter adjustments also replicate the self-assembly of other di- and tripeptides and coassemblies. Furthermore, our analysis uncovers pivotal insights for enhancing the performance of MARTINI in modeling short peptide self-assembly. Specifically, we identify issues stemming from overestimated hydrophilicity arising from charged termini and disruptions in π-stacking interactions due to insufficient planarity in aromatic groups and a discrepancy in intermolecular distances between this and backbone–backbone interactions. This investigation demonstrates that strategic modifications can harness the advancements offered by MARTINI 3 for the realm of short peptide self-assembly.

## Introduction

Over the last few decades, supramolecular peptide self-assembly has emerged as a promising technique for creating new materials with exceptional performance in the fields of nanotechnology and biomedicine.^1−4^ Peptide assemblies have proven to be highly effective as bioactive scaffolds, with successful applications in tissue regeneration both in vitro and in vivo.^5−7^ These materials take advantage of the ability of short, easily synthesized peptides to form well-ordered nanostructures spontaneously through self-assembly. As with proteins, the amino acid sequence of these peptides encodes the final structure, providing control over the morphology, intermolecular cohesion, and surface properties. Even using sequences of only 10 amino acids or fewer, a wide variety of morphologies can be achieved, including fibers, micelles, tubes, ribbons, and sheets.^8−11^ Furthermore, by varying the sequence, materials can range from highly crystalline to lacking intermolecular order, with significant implications for their function.^7,12^ The properties of the structure’s surface are critical for determining its interaction with other biomolecules and cells, as well as other material properties such as viscosity.^5,13−15^ Therefore, the rationalization of the connection between the sequence and structure has become a key priority in the field with the goal of designing materials with on-demand properties.

Understanding the influence of sequence on the properties of the material has consistently been the focus of numerous experimental studies. Most of these works focus on small variations, such as local mutations in the sequence, assessing how different material properties change upon modifying the amino acid’s nature (hydrophobic to hydrophilic, aliphatic to aromatic, or charge) or size.^9,16−18^ Such studies have shown that even minor sequence variations can trigger critical transformations, such as drastic changes in the dimensionality of the formed structures.^8^ Self-assembly is so sensitive to the sequence that isomers with the same amino acid composition can undergo changes due to variations in their positions in the sequence.^19−22^ The sequence length of repeat structural motifs has also demonstrated significant potential for modifying material properties, including intermolecular order, interaction with other structures, and the formation of superstructures.^23,24^ Moreover, even changes to the chemical nature of the C-and N-termini can lead to alterations in the supramolecular order.^25,26^ Recently, researchers have expanded the library of available materials and properties by considering not only the natural L-form of amino acids but also their mirror image, D-forms, providing interesting control over the materials’ behavior within biological media.^27−30^

Despite the numerous advances that have led to the development of a large library of peptide-based supramolecular materials for various applications, only a few design principles can be considered general. Understanding sequence dependence beyond specific instances is limited and often reliant on serendipity, making it challenging to extrapolate. The use of molecular dynamics (MD) simulations has shed some light on this matter. All-atom (AA) models have allowed the study of supramolecular structures at the molecular level, revealing the role of the interactions involved in self-assembly.^26,31−33^ This approach has been extensively applied to assess the stability of different supramolecular arrangements, evaluating, in some cases, the impact of different amino acids on it.^34−37^ In certain cases, the combination of these simulations with experimental results has enabled researchers to construct highly detailed models of supramolecular arrangements. These models have been employed to investigate the hierarchical self-assembly of these materials and the twisting of the structures, among other aspects.^26,38−41^ AA-MD simulations can also account for differences in chirality to assess their effect on self-assembly, as well as how this, and other effects, are influenced by conformational changes induced by charge or sequence variations.^28,30,42^ Moreover, the high resolution of these methods has enabled them to estimate secondary structure content and predict the corresponding spectra for comparison with experimental results.^43−45^ Such detailed models have also demonstrated their potential to reveal other important factors, such as the influence of including functional epitopes and the effect of the ions and other environmental conditions on the resulting structure.^46−48^

In some cases, AA-MD simulations are time-consuming and limit studies to short times or small systems. Additionally, they usually require a high level of knowledge to construct initial configurations, as they often cannot model the entire self-assembly process except in very limited cases.^49^ Therefore, in order to perform on a large scale, both in terms of size (≥10^2^ molecules) and time (≥10^2^ ns), coarse-grained (CG) models have gained popularity. These models sacrifice some of the atomistic detail to expedite simulations, often more than 1000 times. CG models have successfully replicated the formation of self-assembled structures, including micelles, fibers, and tubes,^50,51^ even under constant pH conditions.^52^ Probably, the MARTINI force field is the most widely employed in the field of supramolecular peptide self-assembly due to its exceptional ability to reproduce various features of these materials.^53^ Initially developed to model a different type of supramolecular system, cell membranes,^54^ its extension to proteins unveiled a great prospect to simulate peptide-based self-assembled systems.^55^ This force field models every four heavy (non-hydrogen) atoms with a single CG bead, using a 4-to-1 mapping, including water, where every four molecules are replaced by a single bead. It can also be a 3-to-1 or even 2-to-1 mapping in the case of aromatic rings. The force field has been able to model the formation of assemblies from scratch (molecules randomly oriented and fully solvated), minimizing the bias introduced when initial structures are required. This contrasts with simulations of proteins, where the secondary structure is an input and the tertiary structure requires additional elastic bonds.^56^ Initially, this same procedure was followed, enforcing the β-sheet ordering of an amyloid fibril forming decapeptide to reproduce the nucleation and growth of the fibers.^57^ However, in the same year, Frederix et al. utilized it to reproduce the formation of tubes by the dipeptide diphenylalanine (**FF**).^58^ They leveraged the computational efficiency of the MARTINI model to screen all possible dipeptides and, in a subsequent study, tripeptides.^59^ Using this approach, they not only reproduced the structures formed by known examples but also employed them to discover four new self-assembling tripeptides. The same method, using the aggregation propensity (AP) to quantify self-assembly tendencies, was later employed for the screening of alkylated dipeptides.^60^ In addition to predicting self-assembling sequences, the comprehensive analysis of the results allowed the researchers to establish dependences in the preferred composition and positions of each amino acid, leading to the formulation of design rules. Screening of longer or more complex peptides has relied on the combination of CG-MD simulations with machine learning approaches.^61−63^ This success has led to the use of the MARTINI model to evaluate structure formation by systems designed for specific experimental purposes.^64−66^ This force field has also demonstrated its potential in complex systems, effectively modeling the structures formed when different building blocks are combined,^67,68^ even with varying ratios.^69^ This has encompassed the development of tools to analyze the precise interplay of the monomers in the coassemblies, enabling the distinction of various types of interplays.^70−72^ Moreover, certain studies have employed the MARTINI force field to examine the different phases that emerge in assembled systems,^73,74^ as well as the mechanisms and thermodynamics of self-assembly.^75,76^ Recent efforts have showcased the potential of this force field to contribute to the field of supramolecular peptide assemblies beyond structure prediction. For instance, we have explored the sequence-dependent formation of superstructures, understanding how sequence, conformation, and charge influence them and how filament interactions differ between superstructures and gels.^14^ The study of sequence-dependent interfilamentous interactions has aided in optimizing the experimental conditions for three-dimensional (3D) printing of these materials.^13^ In moving toward using this method to design materials with specific properties, we have succeeded in uncovering the role of molecular motion within the assemblies in the bioactivity of functionalized peptide-based materials with the assistance of MARTINI MD simulations.^7^ We developed a computational approach to screen sequences based on this mobility.^6^ Therefore, we believe that these CG approaches hold significant potential for designing functional supramolecular assemblies with tailored properties.

MARTINI has recently released its third version, which features a higher number of bead sizes and a much more complex interaction matrix, doubling the number of interaction levels. This has significantly improved the level of detail in the model for describing molecules and their interactions.^77,78^ To achieve a more accurate representation of molecules, MARTINI has adopted a “size-shape concept,″ which involves modeling the actual volume and shape of the molecule based on atomistic models. The model can reproduce protein–ligand interactions, while its coarse-grained level still allows for high-throughput screening.^79^ Moreover, the inclusion of Go̅-like parameters to replicate protein flexibility has opened up new possibilities in this field. In addition, the new version of MARTINI includes an approach for constant pH simulations.^80^ These new features hold great promise for the field of supramolecular assemblies, provided we strive to improve our understanding of their interactions with biological systems.

Despite the potential improvements of this third MARTINI version, van Teijlingen et al. recently observed that these changes have led to the diminished ability of the force field to model short peptide self-assembly.^81^ In this study, they present a clear decrease in the aggregation propensities (APs) of dipeptides. They suggest that the new bead size has an effect on the quality of the stacking and demonstrate how balancing the interaction with the solvent can address the issue of the lowered AP. However, their proposed parameters do not result in the formation of the characteristic tubes for diphenylalanine (**FF**); instead, they lead to the formation of discs.

**Figure 1:**
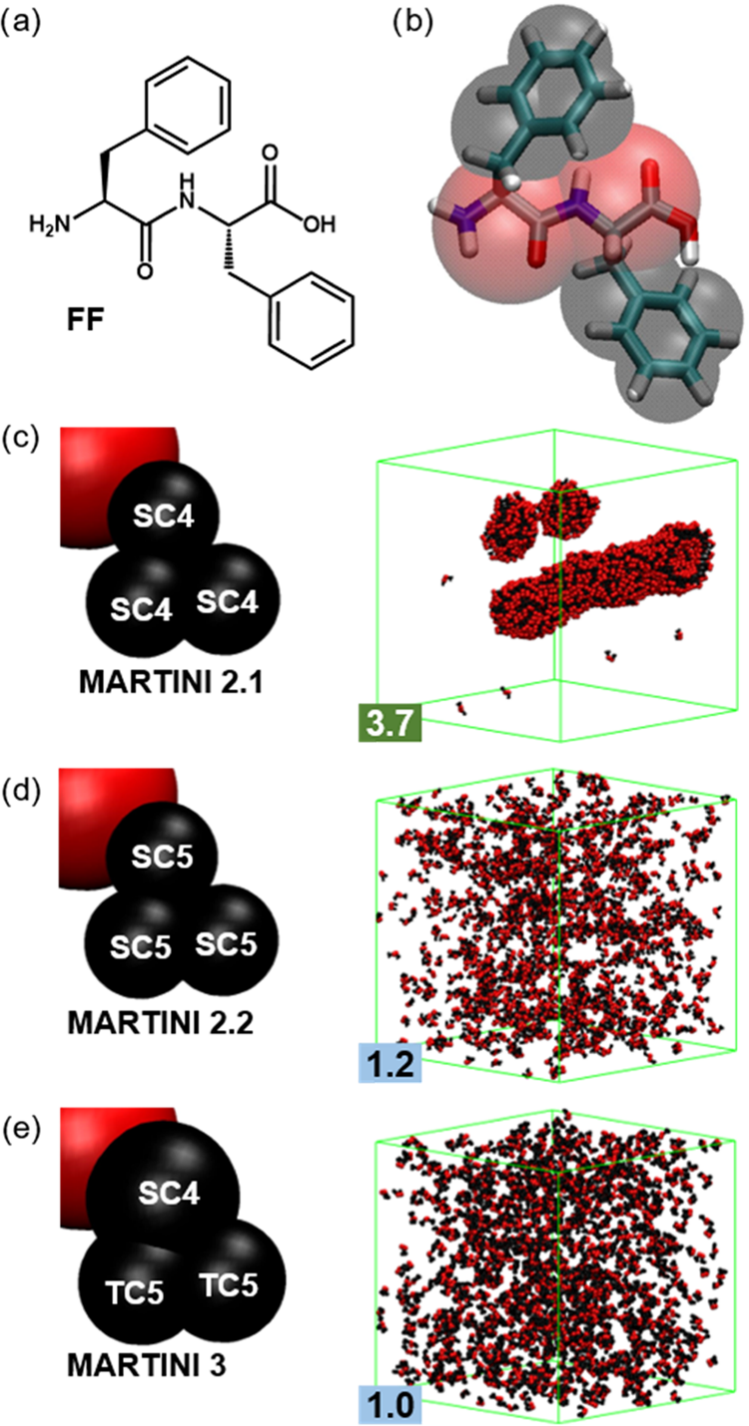
(a) **FF** chemical structure and (b) its general mapping in MARTINI with each F residue represented by a single backbone bead (red) and three side chain beads (black). (c–e) Mapping differences and structures after 5 μs of simulations using 1200 **FF** molecules with versions (c) 2.1, (d) 2.2, and (e) 3 of the MARTINI force field. Water and ions are removed for clarity, and the simulation box is shown in green.

In light of these drawbacks, we assessed the ability of the new MARTINI version to replicate the self-assembly of short peptides in an aqueous solution. We selected diphenylalanine (**FF**, Figure 1a) as the ideal benchmarking candidate, given its propensity to form tubes—a specific shape previously reproduced by the MARTINI model.^58^ Upon evaluation of the new model, we identified certain limitations that we resolved by screening the bead types of the force field. This enabled us to optimize the MARTINI 3 **FF** parameters for the formation of the well-known tubes. Additionally, we analyzed how different simulation and force field parameters affect the reproducibility of the results in different MARTINI versions. Finally, we compared the results obtained using the previous and new MARTINI model across various short peptide assemblers: the dipeptides **FW**, **IF**, **WF**, and **WW**, and the tripeptides **DFF**, **FFD**, **FFF**, **GHK**, **GGG**, **KFD**, **KFF**, **KYF**, **KYW**, **KYY**, and **PFF**, as well as the coassembly between **FFF** and **FF** and **FFD** and **GHK**. Here, **K** stands for lysine, **D** for aspartic acid, **Y** for tyrosine, **W** for tryptophan, **I** for isoleucine, **P** for proline, **G** for glycine, and **H** for histidine.

## Methods

Peptide structures were created in Avogadro and transformed into the MARTINI model using martinize2. We obtained the CG structures and parameters for the MARTINI 2.1, 2.2, and 3 (3001 and two other β) versions of the target peptides. Peptides were simulated in their expected protonated state at physiological conditions; thus, acids deprotonated with a charge of −1 (C-terminus and **D** side chains) and bases protonated with a charge of +1 (N-terminus and **K** side chains). Although previous works have reported on p*K*_a_ shifts upon self-assembly, we do not expect this to be significant for our peptides, given that their zwitterionic nature avoids high concentrations of equal charges.^13^ All systems were set up, simulations were carried out, and analyses were performed using GROMACS 2019.^82^ Initial configuration for each simulation was set up using the *insert-molecules* tool to add a certain number of peptides randomly in a cubic box with a side of 12.5, 17.0, or 24.5 nm. This models an initial fully solvated state that could resemble the effect of solubilizing cosolvents or sonication. Enough Na^+^ or Cl^–^ ions are added when requested to maintain charge neutrality in the system (not necessary for **FF**, as it is with one positive and one negative charge, neutral). No additional ions are included to mimic salts or buffer additives in the experimental systems, following previous protocols.^58,59^ The initial assessment of the ability of MARTINI 3001 to reproduce **FF** self-assembly and the control simulations with the previous releases (MARTINI 2.1 and 2.2) was carried out with 1200 and 1600 molecules in a 24.5 nm cubic box for the final concentrations of 135 and 182 mM, respectively.^58,59^ After this, the first screening of MARTINI 3001 was carried out with 300 and 800 **FF** molecules in 12.5 and 17.0 nm side boxes, respectively, for the final concentrations of 256 and 267 mM, following the 10-fold increment commonly used to speed up self-assembly simulations.^59,83,84^ The second screening was carried out with parameter sets selected from the previous and using the 135 and 182 mM conditions used in the initial assessment. Parameter sets selected in this second screening were subjected to a third screening in which we evaluated the reproducibility of the results in a 17.0 nm side box at different concentrations ranging from 17 to 533 mM (Table 1). The set of dipeptides and tripeptides was simulated using the conditions of Screening Step 0/2. Coassembly simulations followed the procedures from their original works.^69,85^**FFD**:**GHK** coassembly was carried out in a 12.5 nm side box with 300 molecules. For the **FF**:**FFF** coassembly, the systems with **FFF** fractions of 0 (1:0), 0.2 (6:1), 0.7 (9:14), and 1 (0:1) were chosen. With a total number of 1440 **F** residues, these correspond to 720:0, 576:96, 216:336, and 0:480, respectively, simulated in a box of 17.0 nm.

**Table 1:** Simulation Conditions and the Purpose for Each Screening Step for the **FF** MARTINI Parameters

screening step	box side (nm)	no. of molecules	concentration (mM)	replicas	purpose/criteria
0	24.5	1200	135	1	initial assessment of tube formation
		1600	181	1	
1	12.5	300	256	1	screening for AP > 2 and tube-compatible structures
	17.0	800	267	1	
2	24.5	1200	135	1	screening for tube formation
		1600	181	1	
3	17.0	50	17	4	screening for reproducibility in tube formation at various concentrations in medium-sized boxes
		100	33	4	
		200	67	4	
		300	100	4	
		500	167	4	
		600	200	4	
		800	267	4	
		1000	333	4	
		1200	400	4	
		1400	467	4	
		1600	533	4	

Aggregation propensity (AP) was calculated as the ratio between the solvent-accessible surface area (SASA) at the beginning and at the end of the simulations, using the default parameters of the *gmx sasa* tool (0.14 nm solvent probe).^58,59^ In this context, AP = 1 when the final SASA (SASA_f_) matches the initial value (SASA_0_), and AP > 1 when aggregation occurs (*SASA*_*f*_*< SASA*_0_). Typically, aggregated systems are considered with AP ≥ 2. In the context of this study, AP < 1 is not feasible, but it would be, for example, associated with the disassembly of a protein (*SASA*_*f*_*> SASA*_0_). No error is included in the AP determination, as most of them are based only on the last frame of a single simulation. However, it is worth mentioning that the standard deviation across different replicas in the Screening Step 3 is 0.0 within the accuracy used to report the AP. Radial distribution function (RDF) analysis was carried out, selecting the center of mass of each **F** side chain through the last microsecond of the simulation using the *gmx rdf* tool. The temperature of different groups was assessed through the *traj* tool analysis.^86^ Following the procedures of that work, taking into account that for **FF**, *N* = 8, and thus, there are 3*N* = 24 degrees of freedom, but they contain 6 constraints, a 24/18 = 1.333 correction factor is applied for the temperature of the groups. Temperatures were calculated for groups of 200 **FF** molecules, and the average for the last microsecond of the simulation is presented. Structure shape was assessed visually, and all images were rendered using Visual Molecular Dynamics (VMD).^87^ The tube fraction was calculated for a total of four independent simulations at each concentration, and the overall tube formation analysis is the average of the fraction of the different systems considered. The error is the corresponding standard deviation divided by the square root of the number of simulations used minus one.

Interactions used potential shift for Lennard–Jones and the reaction field for electrostatics, with a dielectric constant of 15 and a cutoff of 1.1 nm, and the neighbor list was updated every 10 steps.^88^ Every system was minimized for 5000 steps or until forces converged below 2000 pN. Simulations were run for 50,000,000 steps using a 25 fs time step, corresponding to 5 μs effective time applying the established 4× scaling factor due to CG speed up.^54^ All simulations were run under an NPT ensemble with isotropic conditions using the Berendsen algorithm for the pressure (1 bar, τ_P_ = 3 ps) and V-rescale for the temperature (303 K, τ_T_ = 1 ps).^89,90^ The LINCS algorithm with an order of 4 was employed. However, in order to assess the effect of some parameters in the results, we also carried out simulations with the conditions of Screening Step 0 using particle mesh Ewald (PME) for electrostatics, a frequency of 20 steps to update the neighbor list, a LINCS order of 12, a time step of 20 fs, and the latter two together.^86,91,92^

## Results and Discussion

MARTINI 3 introduces two new bead types with the aim of being more accurate in reproducing the volume and shape of the modeled molecules. So, they introduce the tiny (**T**) beads to represent 2 atoms (1–2 mapping) and keep the small (**S**) beads to represent 3. This new approach modifies the mapping of phenylalanine (**F**). This is still represented by a total of 4 beads, 1 for the backbone and 3 for the side chain. The first side chain bead is kept as an S, like in the previous versions, because it maps three carbon atoms, two belonging to the ring and the C_β_ that links the ring to the backbone. However, the other two beads, as they correspond to two ring carbons each, are mapped by T beads (Figure 1e).

### Screening Step 0

We studied the self-assembly of **FF** with the MARTINI 2 and 3 parameters. We consider the two protein versions of MARTINI 2, 2.1 and 2.2, as well as the two secondary structures most used in simulations of peptide self-assembly, the coil (*C*), where no conformation is favored by any constraint, and extended (*E*), where the parameters introduce constraints to favor the formation of β-sheet arrangements. In MARTINI 2.1, the only difference between *C* and *E* for **FF** is the force constant of the bond between both backbone beads, 400 and 1250 kJ/mol/nm, respectively.^55^ MARTINI 2.2 does not show a difference between both conformations for this peptide length.^93^ The bonded terms of **FF** in MARTINI 2.2 are the same as in MARTINI 2.1 with the *E* secondary structure. However, there is a critical difference between both versions regarding the nonbonded terms of the side chains. Both employ three small apolar beads (**SC**) to represent the side chain, but MARTINI 2.1 uses **SC4** bead types, whereas MARTINI 2.2 uses the more hydrophilic **SC5**. In the case of the backbone, there is no difference, and both of them use the charged type beads (**Q**), **Qd** for the N-terminus and **Qa** for the C-terminus. For the initial screening, we employ simulation conditions that in previous studies successfully led to the formation of **FF** tubes, consisting of 1200 or 1600 **FF** molecules in a 24.5 nm side simulation box.^58^

After 5 μs of simulations, the 2.1 version shows the formation of the characteristic **FF** tubes (Figure 1c).^58^ Although our AP value of 3.7 is above the one presented in that study, 3.2 at 400 ns and 2.7 at 4 μs, these are for the half-sized system with only 300 molecules, and thus, they can be expected to be lower. The substitution of **SC4** beads with less hydrophobic **SC5** in MARTINI 2.2 prevents the aggregation of **FF** molecules, which remain in solution for the whole simulation time (Figure 1d). This lower aggregation propensity of the 2.2 version was first observed by Guo et al. in 2016 but not commented on until van Teijlingen et al. revisited the methodology developed in the Tuttle group for peptide aggregation with the different versions of MARTINI in 2023.^69,81^ Interestingly, the last MARTINI version shows similar performance to 2.2, with no aggregation despite the simulation time being far beyond what is usually required for the self-assembly process, below the microsecond (Figure 1e). In fact, the AP of version 3 is lower than the result in version 2.2 (1.0 vs 1.2). Putting the three versions together (AP_2.1_ (3.7) ≫ AP_2.2_ (1.2) > AP_3_ (1.0)), we can see that the MARTINI improvements in reproducing protein behavior do not reflect in their performance for short peptide self-assembly. Moreover, the earlier iterations of MARTINI 3, *V3.0.B.3.2*, called open β (*v3beta*), and the development version (*v3dev*)^79^ have also been proven unsuccessful in reproducing **FF** tube formation, exhibiting minimal aggregation with AP values ranging between 1.1 and 1.2 (Figure S1). The overestimation of hydrophobic interactions has been commented to favor short peptide self-assembly in the first MARTINI version for proteins, and their correction has diminished the ability of the force field to model the formation of these supramolecular assemblies. The analysis of interaction energies between beads in different versions reveals that from *v2.1* to *v2.2*, the decrease in hydrophobicity arises from an increase in side chain bead interaction with water, rising from 2.7 to 3.1 kJ/mol, approaching the side chain’s self-interaction of 3.5 kJ/mol (Figure S6). In *v3*, the interactions involving charged bead **Q5** remain comparable to previous versions, with *Q5–Q5* at 5.79 and *Q5–W* at 5.64 kJ/mol, while in the previous versions, both were 5.6 kJ/mol. In contrast, interactions of the apolar beads markedly decreased. *SC4–SC4* dropped from 3.5 to 2.35 kJ/mol, along with its corresponding interaction with water, which decreased from 2.7 to 1.80 kJ/mol. Besides the significant drop in the self-interaction of **SC4** beads, it is noteworthy that in previous versions, *SC4–Qa*/*d* interactions were as strong as those of *SC4–W* (2.7 kJ/mol). However, in the last version, *SC4–W* is favored over *SC4–Q5* (1.80 vs 1.48), contributing to an overall increase in the hydrophilicity of the **FF** molecules.

### Screening Step 1

The difference in performance between MARTINI 2.1 and 2.2 suggests that small changes in the hydrophobicity of the side chain can drastically alter their behavior. Furthermore, we must consider that in the original interaction matrix, *SC5–SC5* and *SC4–SC4* interactions are equally attractive and only a lower affinity to the solvent of the latter is enough to drive the mentioned self-assembly differences. Due to this, we carried out a screening of bead types searching for a parameter set that can reproduce the tube formation of **FF** within the MARTINI 3 force field. This screening covers a range of hydrophobicities of the apolar **C** bead types (**C2** to **C5**). Additionally, considering that, as proposed by van Teijlingen et al., the use of different bead sizes to represent the aromatic side chains can disrupt π-stacking interactions, we also screen the use of the same size for the three beads in the rings (**S** or **T**). Looking at the new bead types presented in this version, we decided to consider the use of the *h* bead modification, which accounts for higher self-interaction, to compensate for the potential disruption of the π-stacking. The hydrophilicity of the backbone beads is also screened, considering that the short backbone length can also play a role. For MARTINI 3, the charged beads employed for the backbone go from less to more hydrophilic **Q1** to **Q5**. Lastly, we also check the parameters of open β (*v3beta*) and the development version (*v3dev*), as well as variations with slight changes in hydrophobicity. This screening is carried out using a smaller box size to speed up the process. We employ 300 molecules in the 12.5 nm box used by Frederix et al. before, as well as 800 in 17.0 nm.^59^ This enables us to evaluate the potential effects of the size in systems with similar concentrations, 256 and 267 mM, respectively.

**Figure 2:**
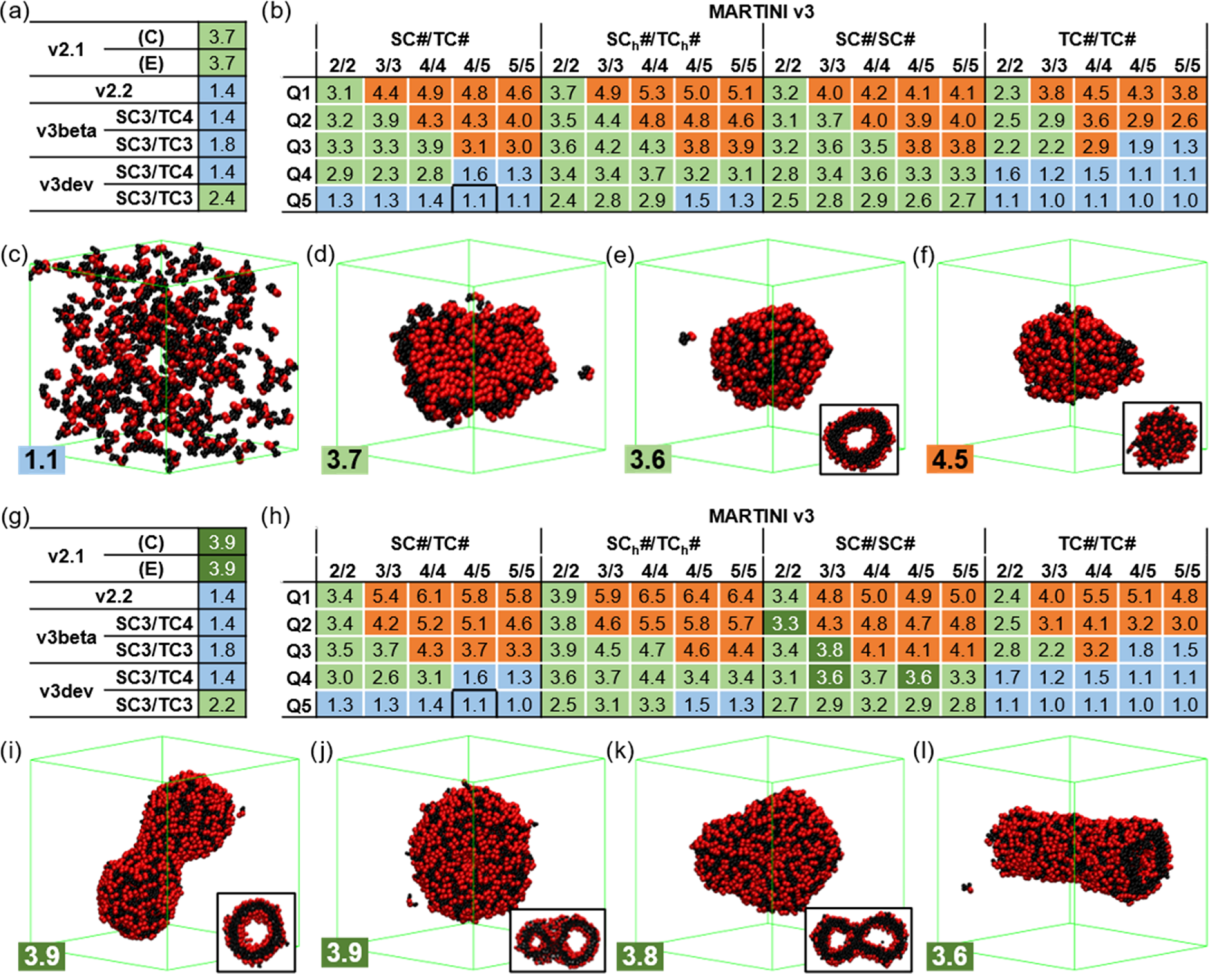
MARTINI 3 Screening Step 1 results for the (a–f) 300 and (g–l) 800 **FF** molecule simulation systems in 12.5 and 17.0 nm side boxes, respectively. (a, g) AP results for MARTINI *v2.1*, *v2.2*, and variations of *v3beta* and *v3dev*, and (b, h) screening of the beads for *v3*. Results are colored according to the structures formed: solution/nonaggregated (blue), solid (orange), tube-compatible (green), and tubes (darker green). Snapshots of (c) **SC4/TC5-Q5** (solution/nonaggregated), (d) **SC4**_**h**_**/TC4**_**h**_**-Q4** (bilayer), (e) **SC4/SC4-/Q4** (vesicle), (f) **TC4/TC4-/Q1** (solid), (i) *v2.1* E (tube), (j) *v2.1* C (tube), (k) **SC3/SC3-Q3** (tube), and (l) **SC4/SC5-Q4** (tube). **FF** molecules follow the color coding in Figure 1, water and ions are removed for clarity, the simulation box is shown in green, and some structures show the cross-section in the inset.

We present the results as a type of phase diagram coloring according to the type of structure obtained from the simulations (Figure 2). In **F** side chain bead nomenclature, the first term corresponds to the bead closest to the backbone and the second term to the other two, which are always equivalent. In MARTINI 3, where also the backbone bead is screened, this is added as a third term. Thus, the original **FF** parameters proposed in *v3* are **SC4/TC5-Q5**. Then, the phase diagram shows: in blue the systems that do not show significant aggregation (Figure 2c), with AP < 2; in orange the solid objects not compatible with the hollow objects expected for **FF** (Figure 2f), with AP values over 2.5, often above 4.0; in green those compatible with the molecular disposition of the tubes, including bilayers and vesicles (Figure 2d,e) that present AP values between 2.2 and 4, except for the *h* beads, which reach values close to 5; and in a darker green those systems that effectively form tubes (Figure 2i–l), which show a better defined AP range between 3.3 and 3.8. The first thing we noticed was that only larger systems were able to form tubes (Figure 2g,h). Not even using *v2.1*, the 300-molecule systems formed tubes (Figure 2a). In general, this size increment enhances AP values, except for those close to 1, the most soluble systems. However, the overall phase diagram is not affected by the size of the system, suggesting that by applying less strict selection criteria, the smaller systems are enough for an initial screening step.

Analyzing the trends in the phase diagram, we see that the hydrophobicity of both the backbone and side chain beads alters the type of structures formed (Figure 2b,h). The bead size or inclusion of the *h* beads is also critical to the structures, tuning the shape of the diagrams. **T**-only side chains present particularly low AP values, whereas *h* beads show a shift to higher APs. Overall, tube-compatible structures are favored by higher hydrophobicities, whereas highly hydrophilic **FF** remains in solution (Figure 2b,h, in blue). There is a clear dependence on the backbone bead, inhibiting the formation of tube-compatible shapes at high hydrophilicities. On the other hand, lowering the backbone hydrophobicity drives the collapse of the molecules into solid objects (Figure 2b,h, in orange). Thus, changes in hydrophobicity have different effects on the backbone and side chains. We can also observe that using **S** beads only or including the *h* beads favors the formation of tube-compatible structures, whereas using **T** beads only reduces the number of these structures. Lastly, **S**-only side chains favor the best results and are the only parameter sets showing tube formation in this type of simulation. In summary, we illustrate with these results that the formation of tube-compatible structures occurs in a certain balance between backbone and side chain hydrophobicity. However, the current **FF** parameters (**SC4/TC5-Q5**) are too hydrophilic (AP = 1.0). The previous versions, *v3beta* and *v3dev*, show slightly better aggregations, with AP values similar to those in *v2.2*, and the enhancement of the side chain hydrophobicity improved these results. However, their values still remain significantly distant from 2, the threshold value established for assembly formation, and are not able to form any tubes (Figure 2).

### Screening Step 2

**Figure 3:**
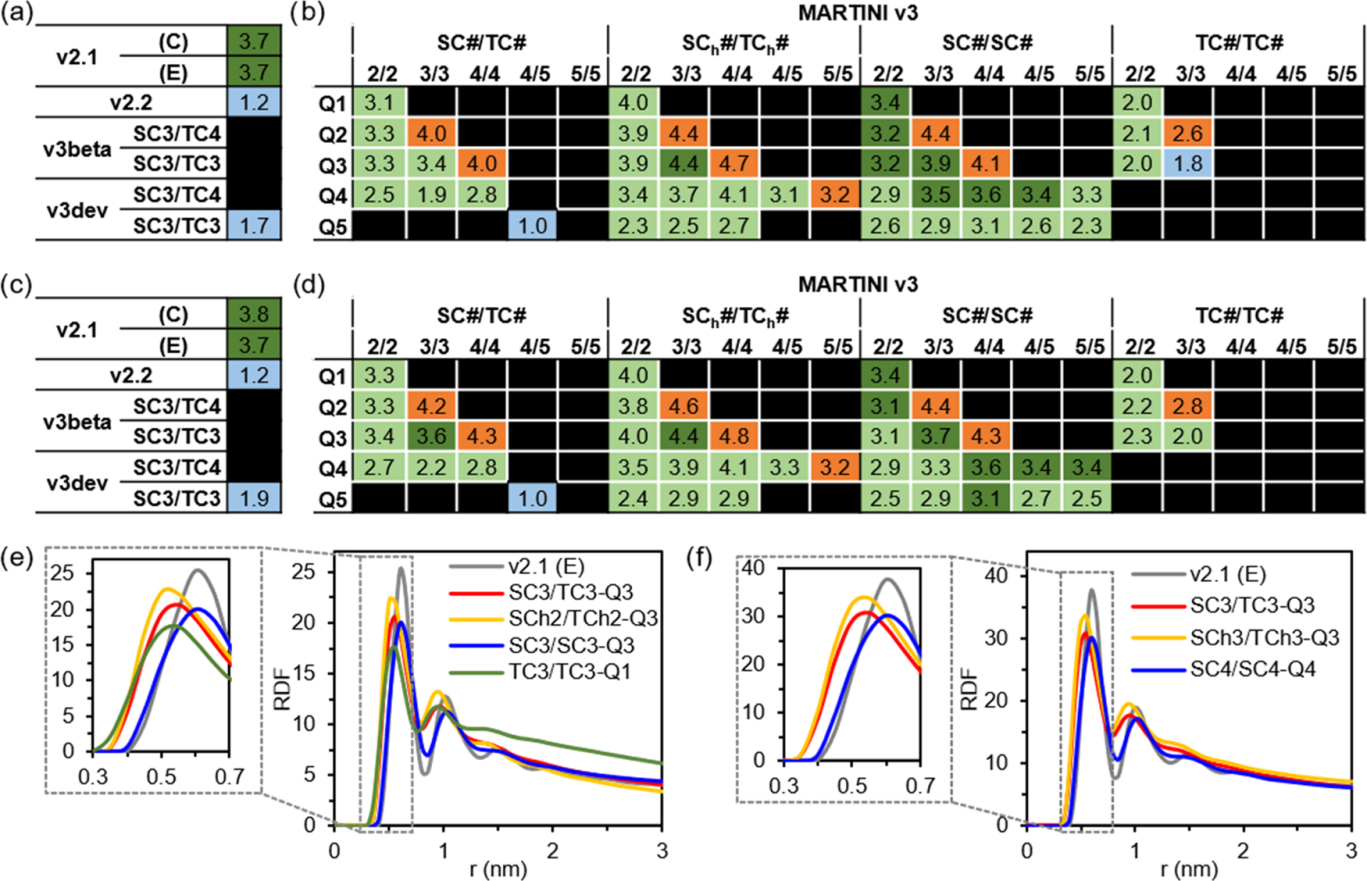
AP results for the MARTINI 3 Screening Step 2, (a, c) including *v2.1*, *v2.2*, and variations of *v3beta* and *v3dev*, and (b, d) the screening of bead types for *v3*, for the (a, b) 1200 and (c, d) 1600 **FF** molecule simulation systems in a 24.5 nm side box. Results are colored according to the structures formed: solution/nonaggregated (blue), solid (orange), tube-compatible (green), and tubes (darker green). Values discarded in the previous screening are shaded in black. Radial distribution function (RDF) graphs of the aromatic side chains (using the center of mass of the three beads) of examples for the different bead compositions in *v3* (e) with a similar AP score from the simulations in Screening Step 1 (Figure 2g,h) and (f) of tube-forming sets from Screening Step 2 (Figure 3c,d). Both include *v2.1 (E)* as a reference and a zoomed region in the inset on the left (dashed in gray).

In the following screening step, we wanted to assess tube formation for all those parameter sets that formed tube-compatible structures in the previous screening and include the MARTINI 2.1 and 2.2 parameters and the current **FF** MARTINI 3 parameters as reference. Given that the box size plays a significant role, we employed the same box size as in the initial assessment. This is a particularly big simulation setup that doubles the previous screening systems and has been used, as mentioned above, to study the morphology of self-assembled structures. Using both 1200 and 1600 molecules in a box of 24.5 nm, we can also get an initial assessment of reproducibility. The results show again the size dependence of the results, with some of the selected parameters giving nonaggregated structures (**TC3/TC3-Q3**, Figure 3b) with 1200 molecules, but not with 1600, and with some others giving formation of solid objects, despite in the previous screening with smaller boxes, they gave tube-compatible structures (orange in Figure 3b,d). These are, in all cases, parameters in the edge of the selection area, and some of them gave, already in the previous screening, ambiguous results among both system sizes. The results show that *v2.1* consistently forms tubes with both secondary structures, while *v2.2* remains too soluble. Additionally, the original MARTINI 3 parameters remain with an AP of 1, irrespective of the simulation conditions. With these results, we also discarded the *v3beta* and *v3dev* options, as well as the **T**-only sets, as none of them showed tube formation in any of the conditions attempted (in the first or second screening). Up to 9 different **S**-only sets are able to form tubes under certain conditions, with 5 of them forming tubes at both concentrations. Only one combined **S/T** set can form tubes with and without the *h*-type beads. However, the latter slightly improves the results, showing tube formation at both concentrations, whereas the former forms tubes with only 1600 molecules. This supports the idea that the different bead sizes cause a certain disruption in the formation of the assemblies. As we mentioned above, van Teijlingen et al. discussed the incompatibility between different bead sizes to form stable π-stacking interactions.^81^ Due to this, we have analyzed the radial distribution function (RDF) of the **F** side chains of certain examples with different bead size distributions that present similar AP values (3.7–4.0) and form tubes (Figure 3e,f, respectively). First, we can observe that there is no clear difference in the trends between the two RDF sets despite the fact that these are different parameter sets and simulation setups. Thus, the RDF depends only on the bead size. *V2.1* and **S**-only sets present the maximum at 0.60 nm (Figure 3e), and all of the sets containing **T** beads are below this (0.50–0.55 nm). In this way, the **T/S**-combined sets present RDF positive values even below the lower limit of the **S**-only π-stacking interactions. This result supports the proposed repulsion between **S** beads due to the forced proximity of well-packed **T** beads in **T/S**-combined sets. According to these results and the fact that the **T/S**-combined parameter sets present a lower tendency to form tubes (Figure 3b,d), the geometrical planarity of the aromatic groups is important to favor π-stacking interactions, often critical in the self-assembly of short peptides. Nevertheless, it is noteworthy that the planar **T**-only sets are even less successful. Their reduced size likely plays a role, as their interaction energies are lower, resulting in diminished differences between self-interactions and interactions with water, thereby affecting the hydrophobic effect. However, upon examining the side chain (Figure 3e,f) and backbone (Figure S2) RDFs, additional challenges become evident. The smaller distances between aromatic groups induced by the **T** beads may be incompatible with the interaction distance of the backbones, leading to significant disruptions in the overall assembly. The analysis reveals that the backbone RDF shows probability densities below 0.1% of the first maximum at 0.4 nm, which is compatible with the stacking distances of *v2.1* and the **S**-only sets, both with values below 5%. In contrast, the **S/T**-combined and **T**-only sets present probability densities of around 30 and 40%, respectively. Thus, in addition to the **T** beads forcing the **S** beads toward unstable distances, there is a discrepancy in interaction distances between the backbone and the side chains or, in other words, between the hydrogen bonding and π-stacking contributions in the assembly of **FF**, posing a potential disruption to the overall assembly, despite the improvements introduced in MARTINI 3 for this type of interaction.^78^

### Screening Step 3

**Figure 4:**
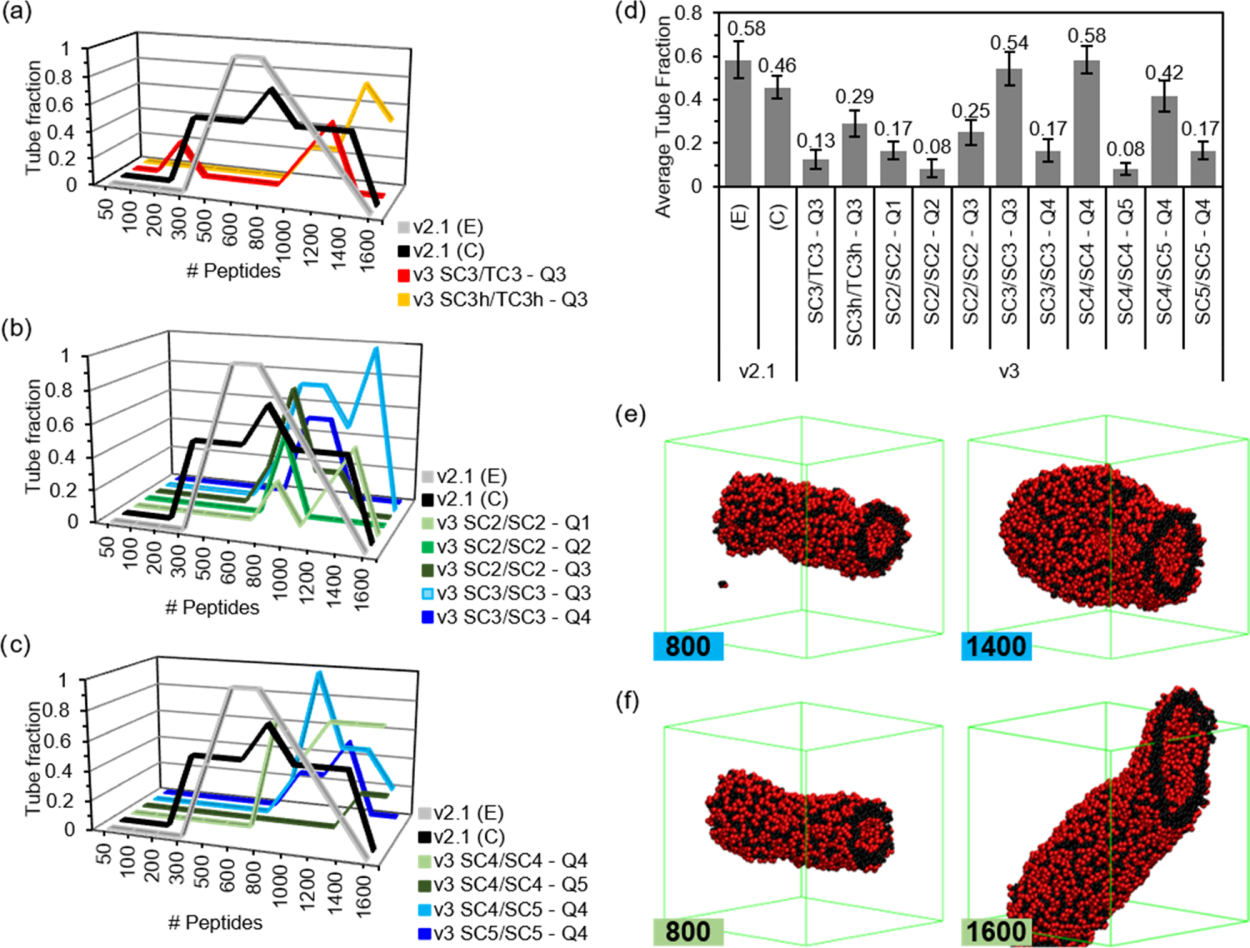
Tube formation reproducibility results from Screening Step 3 showing the tube fraction of *v3* sets, with (a) combined **S/T** sets and **S**-only sets using (b) **C2** and **C3** and with (c) **C4** and **C5**. (d) Average tube fraction for the systems with ≥600 **FF** molecules. Snapshots of the structures formed with (e) **SC3/SC3-Q3** and (f) **SC4/SC4-Q4**, with the number of **FF** peptides indicated in the inset. **FF** molecules follow the color coding in Figure 1, water and ions are removed for clarity, and the simulation box is shown in green.

At this stage, it is clear that the system size affects the structures formed through the self-assembly simulation, with larger systems favoring the more ordered outputs, namely, the tubes. Similarly, seeing that certain parameters can form the tubes under more conditions than others, it seems evident that the reproducibility of the tubes is also a function of the parameters used. Therefore, in order to select among the so-far selected parameter sets, we assessed the tube formation reproducibility. We do this at different concentrations (from 50 to 1600 **FF** molecules), and for this purpose, we select a medium-sized box, 17.0 nm, on each side. The tube fraction is calculated from four independent simulations for each concentration. We observe in the results a high dependence on the concentration that varies strongly with the parameter sets (Figure 4a–c). Even the *v2.1* results show these differences, with a tube fraction of 1 at medium concentrations using the extended (*E*) secondary structure. The distinct concentration dependence observed for conformations *C* and *E* suggests that chain flexibility may also play a role in self-assembly. The more flexible conformation (*C*) exhibits improved reproducibility across various concentrations, while the more rigid one performs best within a narrower concentration range (500–1200 **FF** molecules). However, both the *E* and *C* conformations show fractions of ≥0.5 for the systems between 500 and 1200. Only one simulation among all of the parameter sets (with four replicas each) proposed using MARTINI 3 showed tube formation for systems with fewer than 600 molecules (Figure 4a). So, *v3* parameters require higher concentrations, but most of them fail in the most crowded system of 1600 molecules, similar to the *v2.1* set (Figure S5). Most of the parameters show a high variability but, in general, poor reproducibility. The average tube fraction, calculated using only simulations of at least 600 **FF** molecules, shows that out of the 11 sets, 6 are below 0.2 and only 3 are above 0.4. It is worth mentioning that the trends using all of the systems are the same; only the absolute numbers are lower, and the distance with the MARTINI 2.1 results is enhanced (Supporting Information (SI), Figure S3). Despite the overall bad performance, there are two sets that outperform *v2.1* with the *C* conformation (0.46), and one, **SC4/SC4-Q4**, matches the performance of the *E* conformation with 0.58. These two sets show us how disruptive the enhancement of the hydrophilicity of the **Q** bead can be to the assembly, dropping from 0.54 to 0.17 (**Q3** to **Q4**) and from 0.58 to 0.08 (**Q4** to **Q5**). Additionally, the drop from 0.42 of **SC4/SC5-Q4** to 0.17 of **SC5/SC5-Q4** demonstrates that side chain hydrophilicity also plays a disruptive effect. However, the fact that the best-performing set (**SC4/SC4-Q4**) has an increase in the hydrophilicity of both the side chains (**SC4**) and backbone (**Q4**) over the second one (**SC3/SC3-Q3**) suggests that a certain level of balance between these two is required for proper self-assembling behavior.

In the Supporting Information, we show how the two winning parameter sets are also the closest in AP trends to MARTINI 2.1, which is interesting given the success of this parameter in predicting self-assembly (Figure S4). However, for the systems with tube fractions below 0.4 (Figure 4d), there is no clear correlation. Lastly, the combined **S/T** sets present different concentration dependences, which suggests, again, an effect of the different-sized beads in the aromatic group.

Concerning interactions, the **S/T** set that effectively triggers tube formation (**SC3/TC3-Q3**) necessitates a higher hydrophobicity compared to that of the original *v3* set. This requirement stems from a reduction in the interactions of the **T** side chain beads and the backbone with water, decreasing from 1.36 and 5.64 kJ/mol to 1.12 and 4.99 kJ/mol, respectively (Figure S6). This effect is further magnified by an increase in side chain-charged bead interactions (from 1.48 and 0.98 kJ/mol for **S** and **T** beads, respectively, to 2.16 and 1.45 kJ/mol), thereby promoting aggregation of the **FF** molecules. The incorporation of elevated self-interactions in **SCh3/TCh3-Q3** further amplifies this effect, favoring the formation of tubes (Figure 4d). The **S**-only bead sets exhibit similar trends, reducing the interaction of the charged beads with water while enhancing their interaction with the apolar side chain beads. Consequently, we can conclude that the alterations introduced in MARTINI 3 to the balance of interactions of charged beads with the apolar side chains and water critically impact the model’s ability to accurately replicate the self-assembly of **FF**.

### Simulation Setup Effect

Regarding the system size’s impact, it is important to highlight that the 24.5 nm box used in Screening Step 2 has concentrations (135 and 181 mM) that fall between the concentrations used in the 300 and 600 molecule systems in Screening 3, which were 100 and 200 mM, respectively. However, the larger boxes appear to exhibit better reproducibility in tube formation, as evidenced by their consistency across both concentrations and in line with the results from Screening 1. In Screening 1, the 800 **FF** molecules within 17.0 nm boxes exhibited tube formation in the *v2.1* and three additional MARTINI 3 sets, whereas the 300 molecules within 12.5 nm boxes failed to produce any tubes, even for the former, despite their similar concentrations (267 mM and 256 mM, respectively). Therefore, we can conclude that successful modeling of self-assembled morphologies requires large box sizes. Furthermore, the tube fraction of several studied parameter sets drops significantly at high concentrations (≥1200 **FF** molecules, ≥400 mM). In these excessively congested systems, artifacts formed due to excessive binding across the periodic boundaries of the simulation box (Figure S5). Therefore, we can conclude that particular care must be taken when modeling supramolecular self-assembly with regard to simulation box size and concentration to ensure the reliability and reproducibility of the results. Lastly, it is noticeable that the range of concentrations within which self-assembly exhibits good performance consistently falls between 100 and 400 mM. This range aligns with the 10-fold increment compared to the experimental concentrations typically used when modeling supramolecular self-assembly.^59^

### Validation of the Simulation Parameters

Recent work presented by Thallmair et al. demonstrated the presence of temperature gradients in biphasic water–lipid bilayer systems.^86^ In that study, the use of constraints on the solute molecules led to deviations in the thermostat for different molecular groups, resulting in temperature differences among the components of the same bilayer. As our assemblies are also biphasic and the **F** side chain employs constraints to maintain the geometry of the aromatic ring, they could potentially exhibit similar gradients. Since we do not have multicomponent systems, we analyzed the temperatures of groups of molecules instead, as described in the Methods section. The results revealed no trend among the different models (Figure S7a), and the fluctuations presented are below those shown in the bulk box or in the solvent (Figure S7d). Additionally, tuning the parameters to assess the effect of a different electrostatics algorithm, commonly used in highly charged systems,^91,92^ and a set of parameters demonstrated by Thallmair et al. to minimize these gradients,^86^ we can observe that none of these sets lead to consistent improvements of the temperature fluctuations. Additionally, these parameters have no influence on the AP scores using *v3* despite the recommended time step for this version being employed (Figure S7e).^77^ Therefore, the temperature gradients seem to be below standard fluctuations of the thermostat algorithm, and tuning the simulation parameters does not have a clear effect on them or on the self-assembly behavior of **FF** using *v3*.

### Self-Assembly Validation with Other Di- and Tripeptides

**Figure 5:**
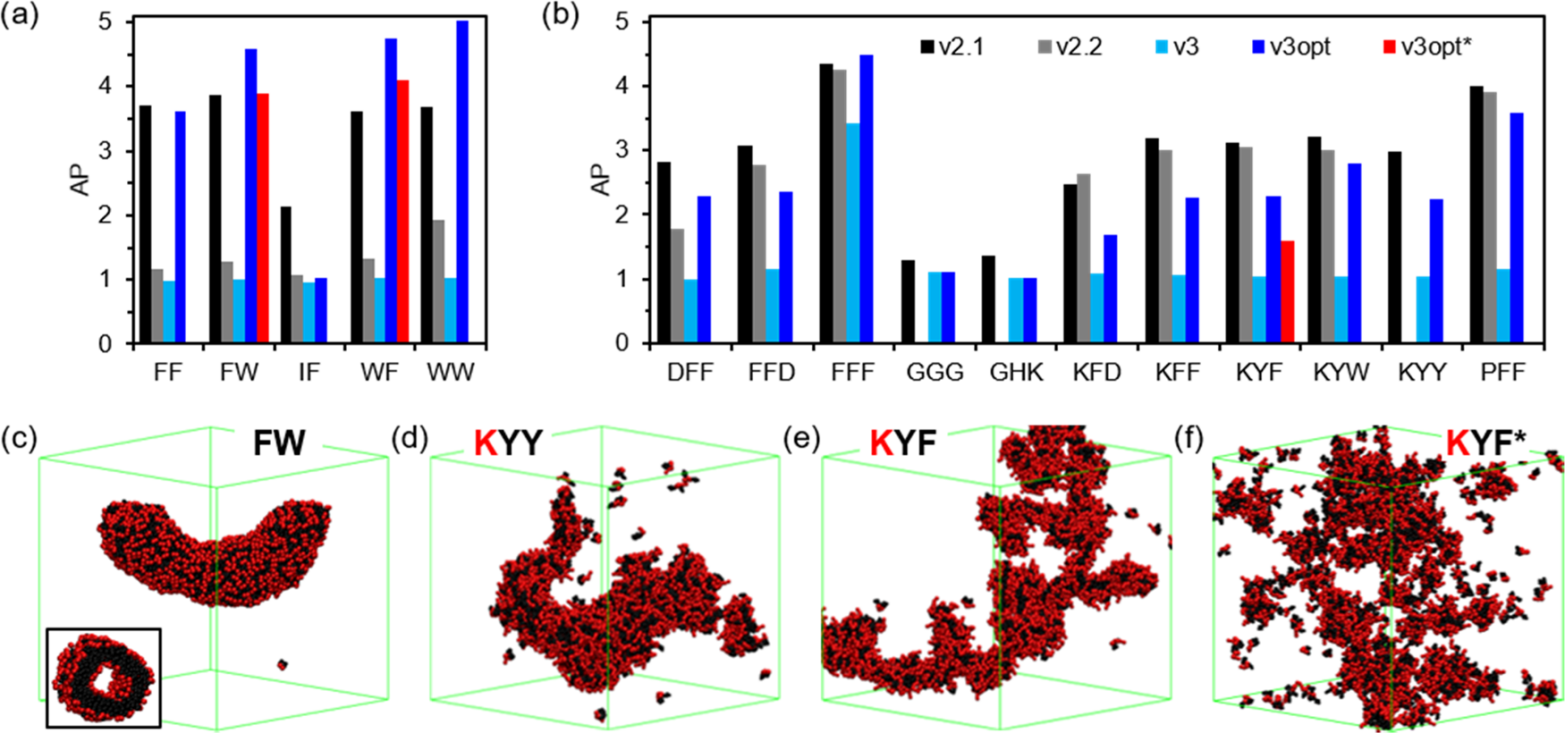
AP scores of the additional (a) dipeptides and (b) tripeptides, using *v2.1*, *v2.2*, *v3*, *v3opt*, and v3opt* (similar to *v3opt* but using *v3* for all of the nonoptimized side chains). Final structures of (c) **FW**, (d) **KYY**, and (e) **KYF** using *v3opt* and (f) **KYF** using v3opt*.

Given the match of the **SC4/SC4-Q4** set with the tube formation tendency of MARTINI 2.1 with the *E* conformation, we decided to assess whether this modification could be enough to fix the self-assembly behavior of other dipeptides and tripeptides. Following the comparison between MARTINI 2.1 and 2.2, and of the different aromatic amino acids within *v2.1*, we attempted a few modifications in these amino acids as well. Our modified **W** and **Y**, as they have additional beads to account for their heteroatoms, employ the same modification of their aromatic hydrophobic beads to **SC4**. Additionally, all of the tested peptides include **Q4** instead of **Q5** for their charged termini, as suggested by our results. We employed the same simulation setup as in the initial assessment, with 1200 or 1600 molecules in 24.5 nm side boxes. In these results, we can observe a significant AP drop from *v2.1* to *v2.2* in dipeptides (Figure 5a), but this difference noticeably decreases for tripeptides (Figure 5b). Instead, *v3* is not able to reproduce any aggregation except in the case of **FFF**. Thus, we see that although both *v2.2* and *v3* exhibit a length-dependent misperformance in reproducing the behavior of short sequences, this issue seems to be more pronounced in the latter version. Therefore, as observed for **FF**, MARTINI 3 di- and tripeptides are unrealistically soluble with AP values of ≈1, except for **FFF**. Nevertheless, we can observe that by introducing our proposed optimization (*v3opt*), the results clearly improve and the AP values increase. The AP of the optimized **FF** is very similar to the reference *v2.1*. The introduction of the **W** modifications in **FW** and **WF** also successfully increases the AP values, in this case, above the reference, suggesting some overestimation of hydrophobicity in the case of these parameters. The results of these two dipeptides with the unmodified **W**, which have AP values closer to those of *v2.1*, may imply that no modification is required for this dipeptide. Nonetheless, the lack of any aggregation in **WW** with the original MARTINI 3 model rejects this option, although it could be related to the hydrophilicity of the backbone beads. The result of **IF** shows that it is not enough to modify only the aromatic amino acid parameters, as the introduction of unmodified aliphatic **I** overrides any improvement of the *v3opt* set (Figure 5a). The results in the tripeptides corroborate this, as only **FFF**, with all of the amino acids modified, reaches AP scores at the level of *v2.1* (Figure 5b). But the modifications clearly improve the results. **Y** seems to be giving improvements similar to **F**, but the hydrophobicity overestimation of **W** makes tripeptides with this amino acid give the best performance. However, future improvements in the rest of the amino acids would make this problematic, leading to excessive aggregations. Lastly, the results on nonaggregating tripeptides, **GGG** and **GHK**, with similar AP values for the *v3* and *v3opt* parameter sets, show that lowering the charged termini hydrophilicity does not lead to any unrealistic aggregation. The results for the 1600 molecules are similar, following the same trends (Figure S8). In summary, the AP results show that there is an improvement with *v3opt* but that **W** presents an overestimated hydrophobicity, and the rest of the amino acids would also require parameter optimization to reproduce the AP behavior of *v2.1*.

Although the AP score provides a good measure of peptide aggregation, we have observed, in the case of **FF**, parameter sets with different APs that lead to tubes or similar AP scores presenting different structures. The AP has not been quantitatively connected to any experimental measure, and thus, using the MARTINI 2.1 results as a reference has limited validity, and it is used only as an initial assessment. Therefore, as for **FF**, we also evaluate the performance of the new parameters in reproducing the formation of structures that have been validated experimentally (Table 2). We must consider that the comparison between experimental and computational results is not always straightforward. Both can have different populations of structures and additional effects, such as experimental drying or computational size of the system, that can alter the output. That is why except for the molecules that remain in solution or the well-ordered tubes and fibers the rest of the structures are compatible with experimentally observed aggregates. Additionally, when referring to some aggregation (some aggr.), it must be stated that this still refers to a state close to molecules in solution. Additionally, some bilayers show preferential growth in one of their dimensions, resembling ribbons that are compatible with the experimentally observed fibers. Continuing with the analysis of the results, the high solubility of *v2.2* and *v3* is totally evident. Although the latter only matches the soluble results, the former can reproduce the self-assembly of some tripeptides. Indeed, some errors of *v2.2* in reproducing the experimental morphology are caused by an excess of order (e.g., **FFF** and **KFD**), similar to *v2.1*. This suggests that the change in behavior for short peptides of the newer MARTINI 2 version is already reduced in tripeptides with respect to dipeptides. However, *v2.1* and *v3opt* are the parametrizations that perform best. Both sets can reproduce the experimental behavior of 4 dipeptides and 10 tripeptides. Actually, *v3opt* only fails to reproduce the behavior of **IF**, undoubtedly due to the nonmodified **I** side chain. Although the formation of the more ordered tubes seems to be favored by modified **W** for **FW** (*v3opt* result), this excess of order may not be consistent with the experimental results for **WF**. In the case of the tripeptides, *v2.1* fails by overestimating the order in **KFD**, whereas the error in *v3opt* is, again, due to a nonreparametrized hydrophobic residue (**P**) for **PFF**. Although the **Y** parameters introduced have not been optimized, they lead to the expected morphologies for **KYY** and **KYF** (Figure 5d,e) and show, as for **W**-containing dipeptides, a better matching than the nonmodified residue for **KYF** (Figure 5f). In summary, although some optimization of the rest of the amino acids would improve the results, *v3opt* can successfully reproduce di- and tripeptide self-assembled morphologies. Indeed, this version seems to have lowered the order overestimation of *v2.1* and has a strong potential to outperform its results with further reparameterization. This aligns with previous reports, highlighting that *v2.1* is deemed “too sticky,” resulting in an overestimation of hydrophobic interactions.^79^ This characteristic likely underlies its efficacy in modeling peptide self-assembly, but it is not unexpected that it may lead to unrealistic overestimation of the order in certain cases. Nonetheless, our proposed set is capable of replicating the positive results of *v2.1* without succumbing to its unrealistic overestimation of hydrophobic interactions.

**Table 2:** Summary of the Morphologies Obtained for the Different Peptides Studied and Their Comparison with the Experimentally Determined Ones^a^

		MD predicted structure			
peptide	experimental structure	*v2.1*	*v2.2*	*v3*	*v3opt*
**FF**	tube, vesicle^94^	tube, vesicle	solution	solution	tube, vesicle
**FW**	tube, aggregate^95^	fiber, solid	solution	solution	tube, vesicle^b^
**IF**	fiber^96^	fiber, solid	solution	solution	solution
**WF**	aggregate^3^	hollow aggr.	some aggr.	solution	tube, vesicle^b^
**WW**	aggregate^3^	fibrillar aggr.	some aggr.	solution	bilayer
**DFF**	aggregate^67^	bilayer	some bilayer	solution	bilayer
**FFD**	fiber^67^	bilayer/ribbon	bilayer	solution	bilayer/ribbon
**FFF**	solid^69^	fiber, solid	fiber	vesicle, tube	solid
**GGG**	solution^59^	some aggr.	-	solution	solution
**GHK**	solution^67^	some aggr.	-	solution	solution
**KFD**	aggregate^59^	fiber	fiber	solution	aggregate
**KFF**	fiber^59^	fiber	aggregate	solution	fiber
**KYF**	fiber^59^	fiber	fiber	solution	fiber, aggr.^b^
**KYW**	fiber^59^	fiber	fiber	solution	fiber
**KYY**	fiber^59^	fiber	-	solution	fiber, aggr.
**PFF**	crystals^59^	solid	solid	solution	tube, vesicle

a The computational results matching (or compatible with) the experimental results are underlined.

b This refers to the structure obtained with v3opt*.

### Coassembly Validation

**Figure 6:**
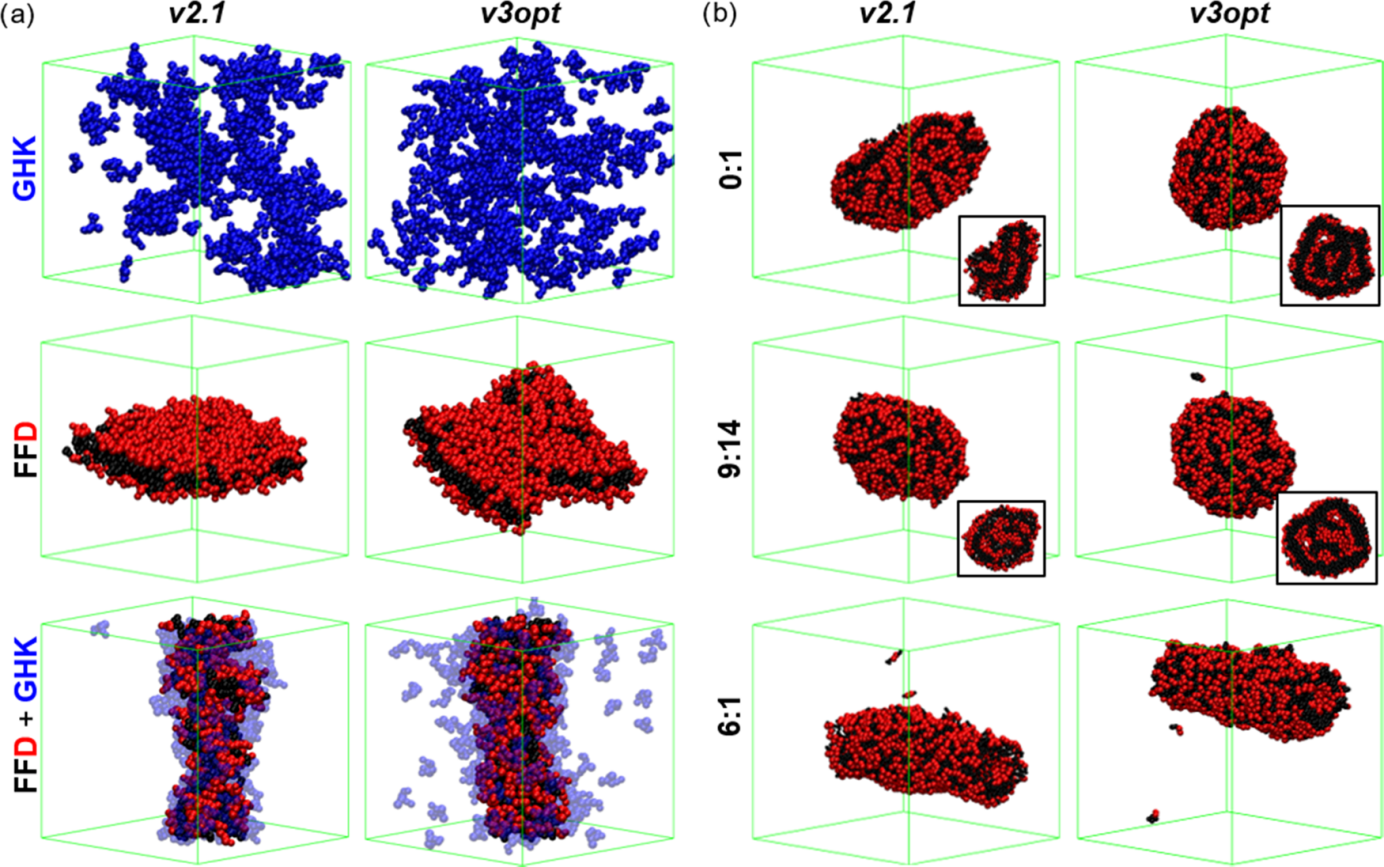
Coassembly results of (a) **FFD** with **GHK**, showing the self-assembled structures of **GHK** and **FFD**, and the coassembly (**FFD**+**GHK**), and (b) **FF** with **FFF** at 6:1, 9:14, and 0:1 (**FFF** only). Some structures show the cross section in the inset.

Given the excellent performance of our parametrization in reproducing the behavior of **FF**, **FFF**, **FFD**, and **GHK**, we also assessed their performance in more complex systems by studying the **FF**/**FFF** and **FFD/GHK** coassemblies. The latter were studied at a 1:1^67^ ratio, while the former at 6:1 and 9:14 ratios, representing the limiting examples of fully hollow and fully solid object formation, respectively.^69^ Following the previous discussion, it is evident that *v3opt* successfully reproduces the expected formation of a ribbon with molecules in a bilayer disposition for **FFD** and the nonaggregation of **GHK** (Figure 6a). Interestingly, **GHK** presents even lower aggregation than that with *v2.1*, which is known to overestimate aggregation (Table 2). This reduced aggregation tendency is also apparent in the coassembly with **FFD**, where a significant number of **GHK** molecules remain in solution. However, similar to when using *v2.1*, *v3opt* demonstrates how the **GHK** tripeptides coassemble onto the **FFD**, enhancing its one-dimensional (1D) character (Figure 6a). In the case of the **FF**:**FFF** coassembly, the results show the formation of hollow structures, specifically tubes, for both versions at a 6:1 ratio (Figure 6b). However, at 9:14 and 0:1 ratios, where **FFF** dominates the assembly, solid objects are formed. Therefore, we can conclude that the proposed optimized parameters for MARTINI 3, *v3opt*, are capable of reproducing more complex behaviors, such as the coassemblies between the proposed dipeptides and tripeptides.

Even though we have demonstrated only the effects of modifying the charged termini and the aromatic amino acids, specifically optimizing only one of them, **F**, our alterations to the MARTINI 3 parameters, *v3opt*, significantly enhance the performance of the original set. This achievement extends to the reproduction of sequence selectivity in the formation of various morphologies, including vesicles, fibers, and tubes. The success of this parameter set is not limited to single-peptide self-assembly systems but has also demonstrated excellent performance in more complex coassemblies involving two components. Therefore, we can conclude that our proposed parameter set enhances the capability of MARTINI 3 to accurately model the self-assembly of short peptides.

We wish to underscore that the proposed set has undergone optimization and validation specifically for replicating the self-assembly and coassembly of short peptides only. This involved enhancing aromatic side chain interactions, crucial for forming the extended stacks inherent to supramolecular assemblies, and reducing the hydrophilicity of the charged C- and N-termini, thereby augmenting aggregation in the presented systems. The significance of this hydrophilicity reduction is particularly pronounced in short peptides, such as dipeptides, where the charged termini constitute the sole backbone beads. Considering that his effect is enhanced by the low stability of side chain-charged termini interactions, it is plausible to assume that the impact of termini hydrophilicity diminishes with increasing length of the peptide. While the exact length required to alleviate this solubilizing effect in *v3* remains unclear, our results already exhibit favorable AP values for **FFF**, indicating that the number of **F** residues in this tripeptide is sufficient to promote aggregation, surpassing the opposite effect of the termini. Consequently, we hypothesize that the overall effect may be negligible in sequences below 10 amino acids and certainly not relevant for proteins, which include additional parameters that ensure structural stability. Nevertheless, minimizing errors may not suffice for certain applications of the MARTINI force field demanding heightened precision in supramolecular interactions, such as ligand binding in the proximity of the termini. In such cases, our proposed correction may yield superior results. However, to rigorously validate these hypotheses, further simulations with longer peptides and proteins—whether embedded in lipid membranes or not—are essential to ensure the MARTINI force field accomplishes its intended purpose. Alternatively, forthcoming MARTINI iterations could benefit from an alternative approach in modeling charged termini to alleviate their destabilizing effect. We propose that decentering the charge from these beads using virtual beads could enhance the consistency of the MARTINI force field across different peptide lengths.

## Conclusions

We conducted an evaluation of the MARTINI 3 force field’s performance in simulating the self-assembly of di- and tripeptides, comparing it with previous versions (2.1 and 2.2) for amino acids. Our analysis revealed that the initial version, *v2.1*, outperformed the others, while the latest version, *v3*, exhibited an inability to replicate the self-assembly behaviors observed in both dipeptides and tripeptides. Additionally, *v2.2* demonstrated diminished performance in dipeptide simulations yet showcased improved outcomes for tripeptide assemblies. Consequently, the iterative refinements made to the MARTINI force field, while enhancing its capacity to emulate protein behavior, have inadvertently diminished its effectiveness in capturing the self-assembly characteristics of short peptides. This trend appears to be associated with the peptide length that has further deteriorated across successive versions.

Motivated by the potential of MARTINI 3, we conducted an exploration of multiple parameter sets to investigate their impact on the force field’s efficacy in reproducing the formation of tubes by dipeptide **FF**. Our investigations into the hydrophilicity variations of charged termini revealed that this factor influences solubility enhancement and likely underlies the length-dependent discrepancies observed in *v3*. This effect is enhanced by disruptive intermolecular interactions between the charged termini and the aromatic beads of **F** and diminishes as the percentage of charged backbone beads decreases with longer peptide lengths and is likely to be insignificant in proteins. While mitigated through the reduction of hydrophilicity in charged beads and augmentation of side chain hydrophobicity, the overestimated repulsion of charged beads could potentially be mitigated in terms of peptide length dependence by relocating the charge away from the backbone bead center in termini. Furthermore, the utilization of distinct bead sizes within the aromatic ring of **F** critically affects π-stacking interactions. Specifically, the use of tiny (**T**) beads brings small (**S**) beads to unstable distances, thereby introducing packing distances that may be incompatible with those between backbones. In summary, we emphasize the significance of maintaining the planar geometry of aromatic side chains to safeguard the integrity of π-stacking interactions in future MARTINI iterations. Additionally, it is crucial to further investigate the impact of discrepancies in interaction distances between the side chains and the backbones within the assemblies.

While acquiring these findings, we also obtained insights into the influence of box sizes and solute concentrations along with the reproducibility of simulation results. Large boxes (≥17 nm) with elevated concentrations (≥180 mM) favored the self-assembly of **FF** into tubes. However, overly congested systems (>500 mM) encountered difficulties in forming the expected structures using specific parameter sets. This dependence was contingent on the parameter sets, which also exhibited concentration-dependent reproducibility. This underscores the importance of screening multiple simulation setups when investigating supramolecular self-assembling systems to ensure the attainment of reliable results or the need for free energy calculations to build accurate phase diagrams of supramolecular systems. It is worth noting that the concentration range at which the simulations are performed optimally aligns closely with the standard practice of using a 10-fold concentration increment compared to experimental values.

Finally, within this study, we introduce parameters designed to enhance the efficacy of MARTINI 3 for simulating short peptide self-assembly. The proposed parameter set (*v3opt*) demonstrates superior performance compared to earlier MARTINI versions in specific scenarios, underscoring the potential of this new version to increase the simulation accuracy of peptide self-assembly phenomena. Unlike the successful MARTINI 2 version, *v2.1*, our optimized version 3 does not appear to necessitate the overestimation of hydrophobic interactions to model this behavior, thus avoiding the overestimation of order observed in certain examples with the older version. In light of these findings, we suggest that refining the side chain geometry and mitigating the impact of charged termini represent promising next steps for future enhancements to the MARTINI force field. These adjustments hold the potential to establish consistency across various peptide lengths from dipeptides to proteins.

## Data Availability

Additional details including the final structures shown in the different figures in gro format, the mdp and itp files employed, and details on the analysis tools can be found at: https://github.com/isasselli/EvalMARTINI3_ShortPeptides.

## Supporting Information Available

Additional structures from Screening Steps 0 and 3; RDF of the backbone beads; average tube fraction and AP of all of the systems in Step 3; energetic terms of the different **FF** parametrizations; temperature differences analysis; and AP of the other dipeptides and tripeptides in the higher concentration system (PDF)
