# Supplementary material for: Assessment of the MARTINI 3 Performance for Short Peptide Self-Assembly
Source: J Chem Theory Comput. 2023 Dec 19;20(1):224–38. doi: 10.1021/acs.jctc.3c01015 (PMC10782451; doi:10.1021/acs.jctc.3c01015)
Supplement: Supplementary file 1 — ct3c01015_si_001.pdf [file ct3c01015_si_001.pdf]

Supporting Information for:

Assessment of the MARTINI 3 Performance for Short Peptide Self-Assembly

Ivan R. Sasselli,[1,2,]* *Ivan Coluzza.*[3,4]

[1]Centro de Física de Materiales (CFM), CSIC-UPV/EHU, Paseo Manuel de Lardizabal 5, 20018 San Sebastián, Spain.

[2]Center for Cooperative Research in Biomaterials (CIC biomaGUNE), Basque Research and Technology Alliance (BRTA), Paseo de Miramón 182, Donostia-San Sebastián, 20014, Spain

[3]Ikerbasque, Basque Foundation for Science, Plaza de Euskadi 5, Bilbao, 48009, Spain

[4]Computational Biophysics Lab, Basque Center for Materials, Applications and Nanostructures (BCMaterials), Buil. Martina Casiano, Leioa, 48940, Spain

AUTHOR INFORMATION

Corresponding Author

*i.sasselli@csic.es

KEYWORDS Molecular Dynamics, Coarse-Grained, Simulations, Supramolecular, Nanotechnology.

Figure S1. Additional structures from Screening Step 0 3

Figure S2. Radial distribution function (RDF) graphs of the backbone beads 4

Figure S3. Average Tube fraction for all the systems (50 – 1600 **FF** molecules)......................... 4

Figure S4. AP as a function of the number of peptides 4

Figure S5. Structures formed in Screening Step 3 with 1600 FF molecules 5

Figure S6. Epsilon interaction terms relevant for the self-assembly of **FF** 5

Figure S7. Maximum temperature differences and AP scores of the additional simulation parameters 6

Figure S8. AP scores from the systems formed by 1600 molecules of the additional di- and tripeptides... 7

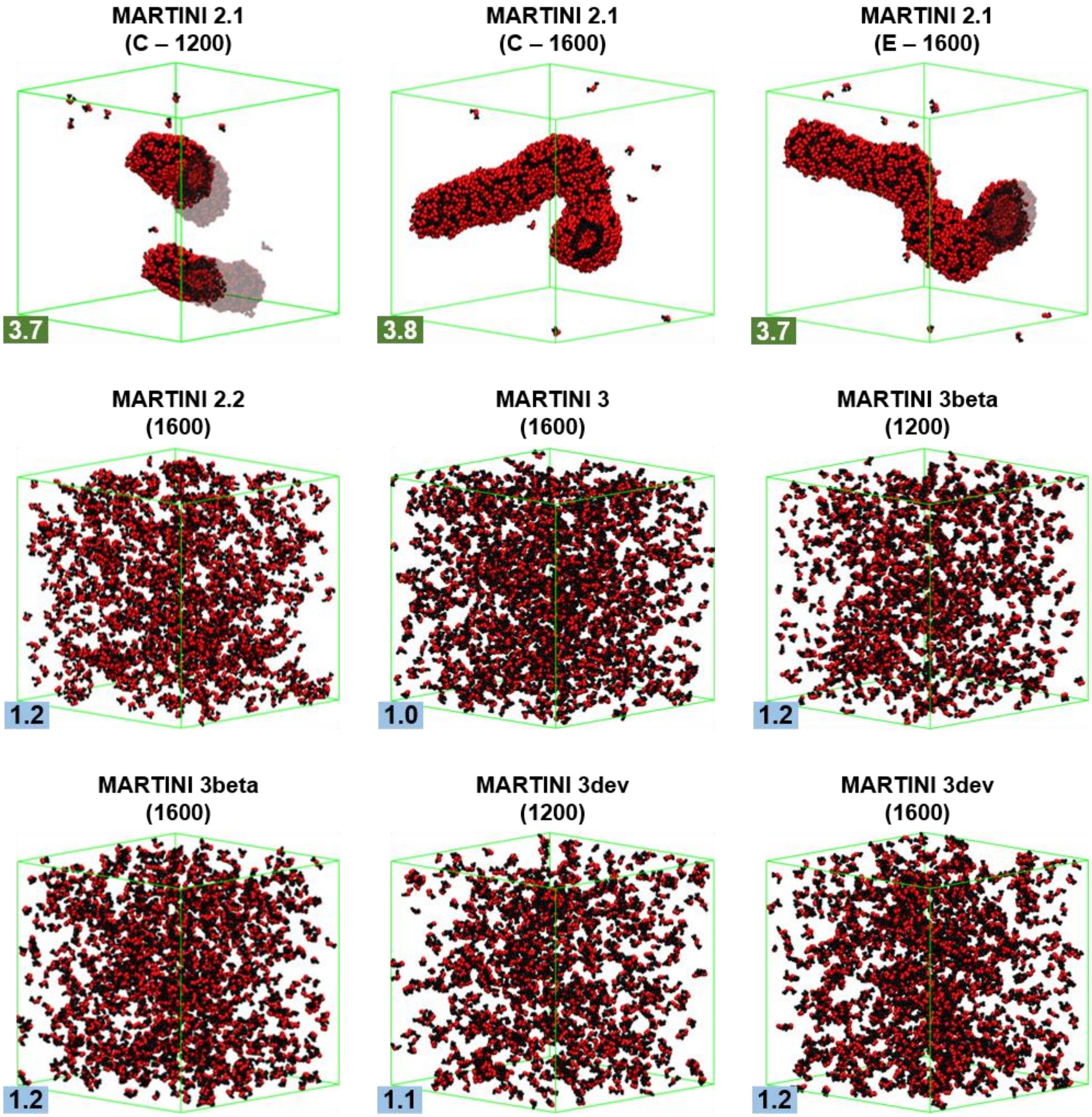

Figure S1. Additional structures from Screening Step 0 (Figure 1). Structures formed through the simulations using the Martini 2.1 (C), 3beta, and 3dev with 1200 molecules, and Martini 2.1 (C), 2.1 (E), 2.2, 3, 3beta, and 3dev with 1600 **FF** molecules.

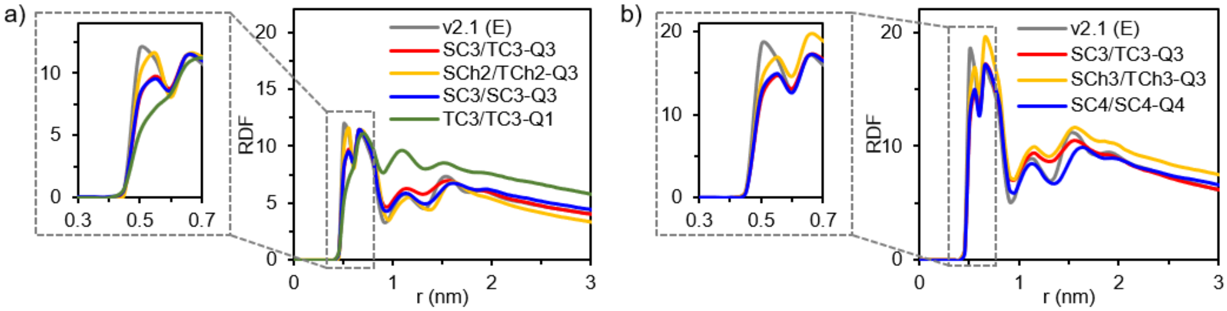

Figure S2. Radial distribution function (RDF) graphs of the backbone beads of **FF** (using the center of mass of both beads) of examples for the different bead compositions in *v3* (a) with a similar AP score from the simulations in Screening Step 1 (Figure 2g-h) and (b) of tube-forming sets from Screening Step 2 (Figure 3c-d). Both include *v2.1 (E)* as a reference and a zoomed region in the inset on the left (dashed in grey)

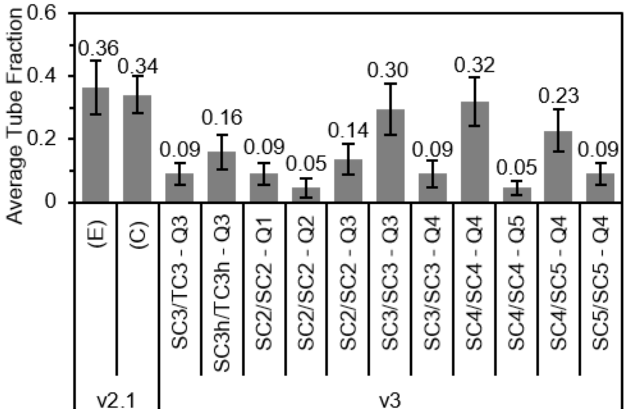

Figure S3. Average Tube fraction for all the systems (50 – 1600 **FF** molecules).

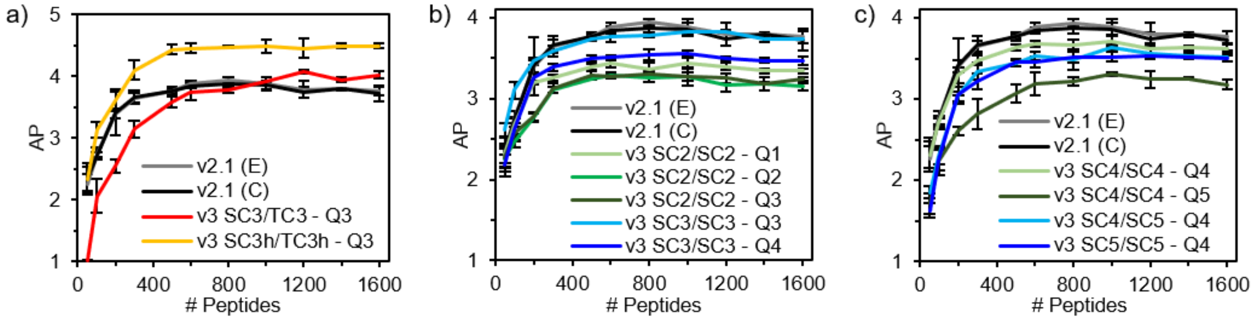

Figure S4. AP as a function of the number of peptides with (a) combined **S/T** sets and **S**-only sets with (b) **C2** and **C3** and with (c) **C4** and **C5**.

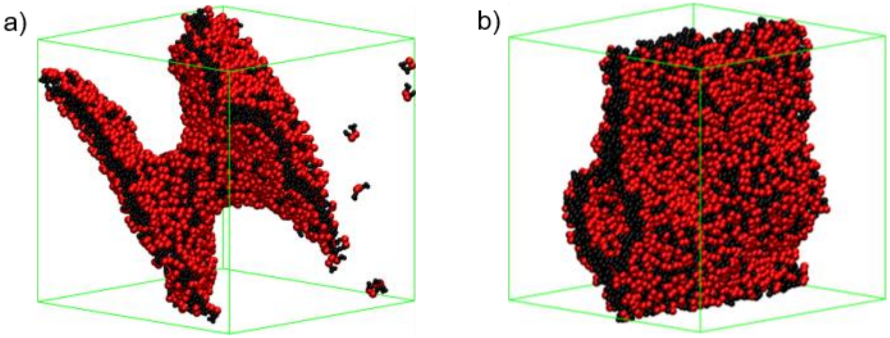

Figure S5. Structures formed in Screening Step 3 with 1600 **FF** molecules using (a) *v2.1* (E) and (b) **SC3SC3-Q3**.

v2.1

	SC4	Qa/d	W (P4)
**SC4**	3.5	2.7	2.7
**Qa/d**		5.6	5.6

v2.2

	SC5	Qa/d	W (P4)
**SC5**	3.5	3.1	3.1
**Qa/d**		5.6	5.6

SC3/SC3-Q3

	SC3	Q3	W
**SC3**	2.35	2.16	1.80
**Q3**		4.73	4.99

v3opt

	SC4	Q4	W
**SC4**	2.35	1.93	1.80
**Q4**		5.24	5.26

v3

	SC4	TC5	Q5	W
**SC4**	2.35	1.91	1.48	1.80
**TC5**		1.51	0.98	1.36
**Q5**			5.79	5.64

SC3/TC3-Q3

	SC3	TC3	Q3	W
**SC3**	2.35	1.91	2.16	1.80
**TC3**		1.51	1.45	1.12
**Q3**			4.73	4.99

SCh3/TCh3-Q3

	SCh3	TCh3	Q3	W
**SCh3**	2.60	2.11	2.16	1.80
**TCh3**		1.77	1.45	1.12
**Q3**			4.73	4.99

Figure S6. Epsilon interaction (kJ/mol) terms relevant for the self-assembly of **FF** in water for the models *v2.1*, *v2.2*, *v3*, and some MARTINI 3 sets: **SC4/TC5-Q5** (*v3*), **SC3/TC3-Q3**, **SCh3/TCh3-Q3**, **SC3/SC3-Q3**, and **SC4/SC4-Q4** (*v3opt*).

a)

# peptides	v2.1 (C)	v2.1 (E)	v2.2	v3 dev	v3 beta	v3 standard	v3 opt
1200	1.6	0.7	2.0	0.4	1.9	2.5	0.6
1600	1.9	1.4	1.4	1.0	1.9	1.4	1.6

b)

# peptides	v3					
	standard	PME	nstlist	LINCS	timestep	LINCS-timestep
1200	2.5	2.3	1.3	3.3	1.6	1.3
1600	1.4	2.1	1.3	1.4	1.5	1.9

c)

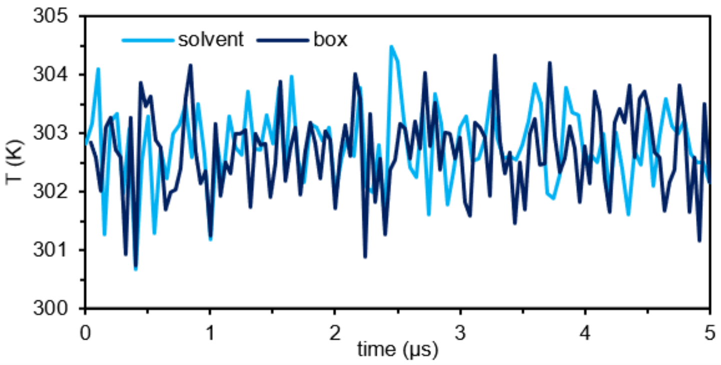

d)

v3 standard 1200	
solvent	box
3.8	3.6

e)

# peptides	v3					
	standard	PME	nstlist	LINCS	timestep	LINCS-timestep
1200	1.0	1.0	1.0	1.0	1.0	1.0
1600	1.0	1.0	1.0	1.0	1.0	1.0

Figure S7. Maximum temperature differences (T_{max}-T_{min}) across the 200 **FF** molecule groups for (a) the different MARTINI models in Screening Step 0 with 1200 and 1600 **FF** molecules, and (b) for *v3* with the additional parameters mentioned in the Methods section. (c) Temperature fluctuations through the 5 μs of simulations and (d) maximum fluctuations through the last microsecond of the solvent and box in the 1200 **FF** simulation using the *v3* **FF** and the standard simulation parameters. (e) AP scores of the additional simulation parameters. (a, b, d) Maximum temperature differences are colored red if higher than 3.0, yellow if between 2.0 and 3.0, light blue

if between 1.0 and 2.0 and dark blue if below 1.0. (e) AP results are colored according to the structures formed, which is blue, corresponding to solution/non-aggregated, for all of them.

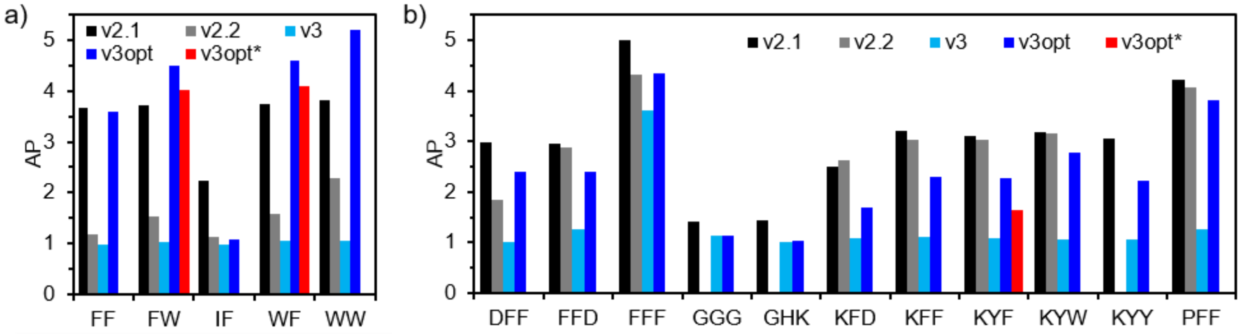

Figure S8. AP scores from the systems formed by 1600 molecules of the additional (a) dipeptides and (b) tripeptides, using the *v2.1*, *v2.2*, *v3*, *v3opt*, and *v3opt** (similar to *v3opt* but using *v3* for all the non-optimized side chains).
